# Editorial: Beyond conventional biomarkers: unlocking immunotherapy response through novel biomarkers or combinatorial approaches

**DOI:** 10.3389/fimmu.2026.2013615

**Published:** 2026-10-13

**Authors:** Ünal Metin Tokat, Nalan Akgul Babacan, Mutlu Demiray

**Affiliations:** Precision Oncology Center, Medicana Health Group, Istanbul, Türkiye

**Keywords:** immunotherapy, immunotherapy resistance, immune checkpoint inhibitors (ICIs), precision immuno-oncology, predictive biomarkers, combination therapy, liquid biopsy (LB), digital pathology

Immune checkpoint inhibitors (ICIs) have reshaped therapeutic landscape in oncology, yet primary and acquired resistance limit their clinical benefit, especially in patients without established biomarkers such as microsatellite instability (MSI) or high tumor mutational burden (TMB-H). This requires us to overcome our reliance on these isolated parameters and decipher the biological mechanisms of therapeutic failure. Our Research Topic, in partnership with *Frontiers in Immunology* and *Frontiers in Oncology*, collectively focuses on the identification of emerging immunotherapy biomarkers to enhance patient stratification and novel modalities for treatment optimization.

## Systematic overviews and multi-omic frameworks

Three reviews in this research topic outlined the current challenges in the field. Across 15 studies, Ismail and Hassan found that ICI combinations could prolong survival but increased severe immune-related adverse events, which routinely exceed those reported in the clinical trials. Furthermore, conventional efficacy biomarkers do not reliably predict toxicity. Kim et al. examined the immunotherapy response and resistance biomarkers in glioblastoma (GBM), proposing liquid biopsies, spatial multiomics and radiogenomics to address intratumoral heterogeneity beyond PD-L1 or TMB alone. These approaches should be rigorously tested and systematically validated to change the clinical management of the GBM patients. Alshamlani et al. reviewed 31 studies linking long non-coding RNA signatures to survival and ICI response. Together, these reviews support dynamic, multimodal biomarker development assisted by artificial intelligence, with separate models for benefit and harm.

## Liquid biopsies and systemic indices

Several articles in the topic explored accessible circulating measures. Xie et al. introduced the Lung Cancer Immune Prognostic Score (LCIPS), combining the albumin-to-globulin ratio with monocyte and lymphocyte counts to predict pathological complete response (pCR) and survival after neoadjuvant chemoimmunotherapy in non-small cell lung cancer (NSCLC). This novel tool could complement the conventional markers or offer an insight alone when they are not available. In extensive-stage small-cell lung cancer, Liu et al. assessed ferritin changes alongside inflammatory markers, associating low baseline ferritin and a significant decrease during treatment (high ΔSF) with better chemoimmunotherapy response and progression-free survival (PFS). This cost-effective biomarker panel also bridges iron metabolism and anti-tumor immunity. Wang et al. used neutrophil-to-lymphocyte and platelet-to-lymphocyte ratios in a prognostic nomogram for immunotherapy-treated recurrent or metastatic cervical cancer. This inflammatory signature could be assessed in prospective studies to confirm if we could reliably integrate such combinatorial models into precision immuno-oncology workflows.

Metabolic and hormonal markers could similarly serve as complementary and/or independent predictors for the immunotherapy response. Zhao et al. identified elevated pretreatment cortisol as an independent marker of poorer disease control, PFS and overall survival (OS) during anti-PD-1 treatment for advanced gastric cancer. In a prospective study of first-line chemoimmunotherapy in the same disease, Liu et al. linked lower circulating lactate and pyruvate to better disease control, PFS and OS. The study highlights a novel, liquid biopsy-based approach, shifting focus from traditional tissue markers to metabolic profiling. These observations also motivate studies of iron metabolism and glucocorticoid receptor modulation, without yet establishing either as a therapeutic strategy.

## Cellular and host immune profiling

Circulating immune cells may reveal information missed by soluble markers. In hepatocellular carcinoma, Lu et al. associated IFN-γ-producing cytotoxic lymphocytes (CTLs), but not serum IFN-γ levels, with response and tumor marker decline during PD-(L)1 therapy. Thus, IFN-γ^+^ CTLs could be a new cellular biomarker for predicting ICI efficacy. Schmidt et al. developed a machine-learning signature from peripheral CD8+ T cells in NSCLC; effector memory features, including CD44 and GIMAP4, predict long-term ICI response more accurately than PD-L1 TPS in their cohort.

Longitudinal profiling challenges the assumption that non-response reflects immune inactivity. In the PRIMMO trial, Baiden-Amissah et al. describe concurrent activation and regulation, including CD69, ICOS and Tregs, in nonresponding advanced cervical and endometrial cancers treated with pembrolizumab, radiotherapy and an immunomodulatory cocktail. Longitudinal immune monitoring is required to track this dynamic equilibrium and tailor adaptive combination regimens.

Host genetics adds another perspective and complexity to the prediction of the ICI response. Watanabe et al. demonstrated that HLA-DRB1*04:05, an allele linked to type 1 diabetes, could result in shorter PFS and OS among Japanese patients receiving ICIs, supporting further population-specific immunogenetic assessment.

## Digital pathology and spatial tissue dynamics

Tissue-based approaches could be equally relevant and important in some contexts. In a multicenter study, Chen et al. evaluated stromal tumor-infiltrating lymphocytes, tumor-stroma ratio and tumor budding on routine H&E slides in advanced colorectal cancer (CRC). High lymphocyte infiltration and low stromal content correlated with better objective response and PFS, including in mismatch repair-proficient (pMMR) tumors. Standardizing these cost-effective morphological biomarkers could provide a highly accessible complement to molecular testing, improving patient stratification in real-world immunotherapy settings. Chayen et al. introduced ENLIGHT-DP, which infers transcriptomics from H&E slides in advanced cutaneous squamous cell carcinoma, stratifying PFS and achieving an 84.2% positive predictive value for PD-1 inhibitor response. It outperformed conventional clinical biomarkers in the study, which is promising to bypass resource-intensive genomic testing and streamline personalized immunotherapy stratification in the future. In esophageal squamous cell carcinoma, Wang et al. combined endoscopic and hematological data in an interpretable random-forest/machine learning model to predict pCR after neoadjuvant chemoimmunotherapy. This approach could overcome the limitations of traditional imaging, offering a robust tool for patient stratification.

At finer resolution, Wu et al. combined single-cell and spatial transcriptomics to examine interactions between natural killer T and type 1 helper T cells in CRC. Their 7-gene NTRG score paradoxically associates higher values with greater ICI response but poorer OS, illustrating why treatment benefit and prognosis should not be treated as interchangeable. Such signatures could connect spatial biology with single-sample bulk RNA sequencing.

Other studies in this section connect biomarkers to therapeutic choices. In advanced biliary tract cancer, He et al. utilized DNA damage response alterations and high TMB to distinguish patients benefiting from first-line sintilimab, anlotinib and gemcitabine/cisplatin from those better served by chemotherapy alone. Crossman et al. report that tumor-bound MerTK overexpression in triple-negative breast cancer can support a pro-inflammatory microenvironment and ICI sensitivity irrespective of PD-L1, contrasting with its usual immunosuppressive role.

## Combinatorial salvage strategies

Finally, three case reports explored if combinatorial approaches could enhance immunotherapy response in treatment-refractory setting. Yu and Li described an 85-year-old patient with recurrent MSI-high/mismatch repair-deficient, *BRAF* V600E-mutant CRC treated with pembrolizumab, dabrafenib and trametinib. Severe pyrexia prompted early targeted-therapy discontinuation, but pembrolizumab maintenance sustained a complete radiographic response beyond 26 months. Whether brief MAPK inhibition contributed to immune priming remains a hypothesis.

Wang et al. report durable disease control with an VEGFR TKI plus PD-1 blockade in metastatic *TSC2*-mutant malignant perivascular epithelioid cell tumor (PEComa) progressing on everolimus.

Our group described *PTCH1*-mutant basal cell carcinoma with squamous differentiation that resisted dual ICIs despite ultra-high TMB (Aydın et al.). Vismodegib plus pembrolizumab subsequently produced a durable near-complete metabolic remission. Our work demonstrates that oncogenic pathway-mediated immune exclusion can override high antigenicity, highlighting the necessity of biology-guided combinatorial strategies in biomarker-discordant tumors.

These cases suggest that pathway-driven immune exclusion may limit responses despite favorable biomarkers and justify testing targeted therapy-ICI combinations. However, they cannot explain the contribution of individual drugs.

## Conclusion

Overall, the diverse contributions to this research topic demonstrate that unlocking the full potential of cancer immunotherapy requires moving beyond single, static biomarkers. Whether through minimally invasive liquid biopsies capturing dynamic metabolic and peripheral immune changes, digital pathology inferring spatial and transcriptomic landscapes directly from routine histology, or immunogenetic profiling of host factors, these studies highlight the power of integrative and multidimensional approaches. Furthermore, translational case reports and combinatorial trials demonstrates that understanding oncogene-mediated immune exclusion and strategically targeting it alongside ICIs can successfully mitigate immunotherapy resistance even in biomarker-discordant tumors. Ultimately, transitioning these discoveries from exploratory models into prospective and adaptive clinical trial designs will be critical to establish robust predictive frameworks, uncouple efficacy from toxicity, and deliver truly personalized IO for patients across diverse malignancies.

